# PVP-Assisted Synthesis of Self-Supported Ni_2_P@Carbon for High-Performance Supercapacitor

**DOI:** 10.34133/2019/8013285

**Published:** 2019-11-13

**Authors:** Qian He, Xiong Xiong Liu, Rui Wu, Jun Song Chen

**Affiliations:** ^1^School of Materials and Energy, University of Electronic Science and Technology of China, Chengdu 611731, China; ^2^Center for Applied Chemistry, University of Electronic Science and Technology of China, No. 2006, Xiyuan Ave. West Hi-Tech Zone, Chengdu, China

## Abstract

Highly conductive and stable electrode materials are usually the focus of high-performance supercapacitors. In this work, a unique design of Ni_2_P@carbon self-supported composite nanowires directly grown on Ni foam was applied for a supercapacitor. The Co_3_O_4_ nanowire array was first synthesized on the Ni foam substrate, and the resulting Ni_2_P@carbon nanocomposite was obtained by hydrothermally coating Co_3_O_4_ with the Ni-ethylene glycol complex followed by gaseous phosphorization. We have discovered that the molecular weight of surfactant polyvinylpyrrolidone (PVP) used in the hydrothermal step, as well as the temperature for phosphorization, played very important roles in determining the electrochemical properties of the samples. Specifically, the sample synthesized using PVP with 10 k molecular weight and phosphorized at 300°C demonstrated the best supercapacitive performance among the different samples, with the highest capacitance and most stable cyclic retention. When an asymmetric supercapacitor (ASC) was assembled with this Ni_2_P@carbon sample as the cathode and activated carbon (AC) as the anode, the ASC device showed excellent capacitances of 3.7 and 1.6 F cm^−2^ at 2 and 50 mA cm^−2^, respectively, and it kept a high capacitance of 1.2 F cm^−2^ after 5000 cycles at a current rate of 25 mA cm^−2^. In addition, the ASC could reach a high energy density of about 122.8 Wh kg^−1^ at a power density of 0.15 kW kg^−1^ and 53.3 Wh kg^−1^ at the highest power density of 3.78 kW kg^−1^. Additionally, this device also had the ability to power up 16 red LEDs effortlessly, making it a strong candidate in electrochemical energy storage for practical usage.

## 1. Introduction

In recent decades, energy has been recognized as an increasingly important component for economic development and social activities. The renewable energy storage and conversion systems (e.g., battery, supercapacitor, electrocatalytic device, and fuel cell) need to be better developed so as to deal with the global energy crisis and the associated environmental problems [1–5]. In these systems, the electrode material is undoubtedly a very important aspect in determining the performance of the overall device [6, 7].

For supercapacitors, they can be generally divided into two different types based on how the charges are stored [8, 9]. The first type is usually called electrical double-layer capacitors (EDLCs), which utilize electrostatic interaction between the charges and electrodes for energy storage [3, 9]. The second type is referred to as the pseudocapacitors, which store charges via reversible faradic reactions at the electrode surfaces [10–12]. In either category, the electrode materials have to provide highly efficient kinetics for charge transfer in order to deliver appropriate energy on demand [13, 14]. Because pseudocapacitors rely on redox reactions for charge storage and release, they usually offer higher capacitance compared to EDLCs which mainly employ high-surface-area carbons as the electrode [14]. Due to this distinct feature, semiconductors like metal hydroxides/sulfides/phosphides are usually used as electrode materials in pseudocapacitors, which unfortunately have intrinsically low conductivities [15, 16]. In recent years, self-supported materials, which could be defined as active materials directly grown on the conductive substrate, have emerged to specifically address this issue [17, 18].

Among the myriad of different candidate electrode materials for pseudocapacitors, transition metal phosphides, such as nickel phosphide, possess both metalloid characteristics and better electrical conductivity over oxide and hydroxide counterparts, suggesting their great potential in electrochemical applications [19, 20]. After being intensively researched, it is now realized that the transition metal phosphides demonstrated superior electrocatalytic performance in HER and OER, as well as high-energy storage capacity in a supercapacitor [20–24]. However, to the best of our knowledge, the research on self-supported metal phosphides for supercapacitor application is still in its infant stage, and only very few works were reported [20, 25]. In order to bridge this gap and to better explore the potential of these metal phosphides, we are greatly motivated to explore a facile and cost-effective method to synthesize self-supported transition metal phosphide for high-performance supercapacitor application.

In this work, we firstly synthesized the Co_3_O_4_ nanowires (NWs) on Ni foam as the backbone and then hydrothermally coated Co_3_O_4_ with the Ni-ethylene glycol complex (Ni-EG) with the assistance of polyvinylpyrrolidone (PVP) followed by gaseous phosphorization to obtain a Ni_2_P@carbon nanocomposite [26]. The as-prepared samples contained micrometer-thick films formed on the surface of the Ni foam substrate, with carbon-coated Ni_2_P one-dimensional structures. It is discovered that the molecular weight of the PVP used in the hydrothermal process and the phosphorization temperature have a significant effect on the morphology of the final product. As a result, the Ni_2_P@carbon nanocomposite synthesized using PVP-10k and phosphorized at 300°C exhibited remarkable supercapacitor performance of 13.8 F cm^−2^ at a discharge current rate of 2 mA cm^−2^ and 8.5 F cm^−2^ at a high discharge current rate of 50 mA cm^−2^, and it also delivered a high capacitance of 5.5 F cm^−2^ after 2000 charge-discharge cycles. More significantly, when an asymmetric supercapacitor (ASC) was assembled with the Ni_2_P@carbon sample as the cathode and activated carbon (AC) as the anode, the device showed excellent capacitances of 3.7 and 1.6 F cm^−2^ at 2 and 50 mA cm^−2^, respectively, and it kept a high capacitance of 1.2 F cm^−2^ after 5000 cycles at a current rate of 25 mA cm^−2^. In addition, the ASC could reach a high energy density of about 122.8 Wh kg^−1^ at a power density of 0.15 kW kg^−1^ and 53.3 Wh kg^−1^ at the highest power density of 3.78 kW kg^−1^. Additionally, this device also had the ability to power up 16 red LEDs effortlessly, making it a promising candidate for practical usage.

## 2. Results and Discussion

**Figure 1:**
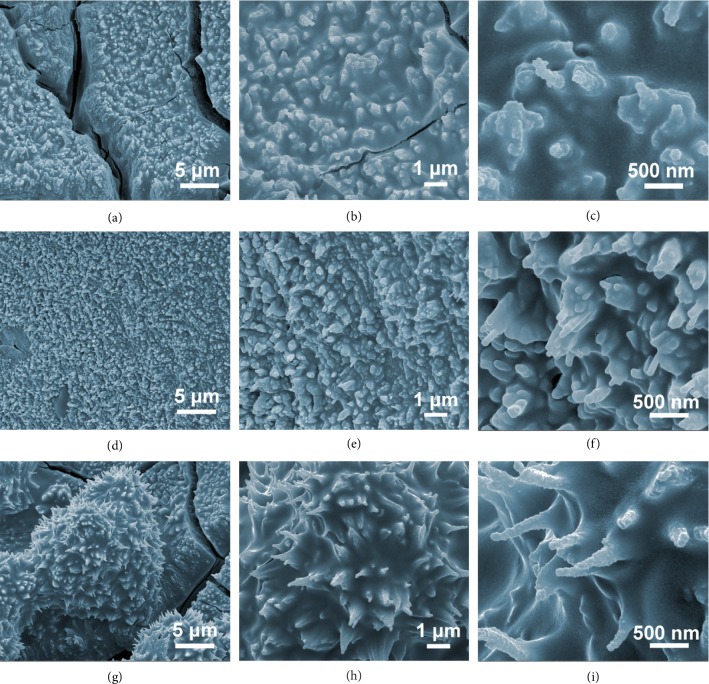
FESEM images of (a–c) NP-10k-T3, (d–f) NP-40k-T3, and (g–i) NP-360k-T3 at different magnifications.

The morphology of the as-prepared samples is investigated with a field emission scanning electron microscope (FESEM). As shown in [Supplementary-material supplementary-material-1], samples of Co NWs, Co_3_O_4_ NWs, and Co_3_O_4_ NWs-Ni all well maintain the configuration of a nanowire scaffold. Subsequently, to investigate morphological differences with adding PVP of different average molecular weights, PVP-dependent experiments are carried out. It can be seen in Figures 1(a)–1(i) that the samples of NP-10k-T3, NP-40k-T3, and NP-360k-T3 synthesized with PVPs of different molecular weights all contain a film-like structure covering the entire surface of the Ni foam substrate. With a closer look (Figures 1(c), 1(f), and 1(i)), it can be observed that countless rod-like particles are embedded inside the film with the tips protruding out from the surface. Additionally, samples NP-40k-T3 and NP-360k-T3 appear to have better-defined one-dimensional constituents compared to NP-10k-T3, suggesting that PVP with higher molecular weight could probably better preserve the nanowires after the gaseous phosphorization. By having such a carbon layer on the surface of the nickel foam with all the active materials embedded inside, it offers a relatively good electrical conductivity as well as a cushion layer that could buffer the volume changes during the charge-discharge cycles. As a result, the contact between the current collector and the Ni_2_P is ensured and the structure stability is well maintained.

**Figure 2:**
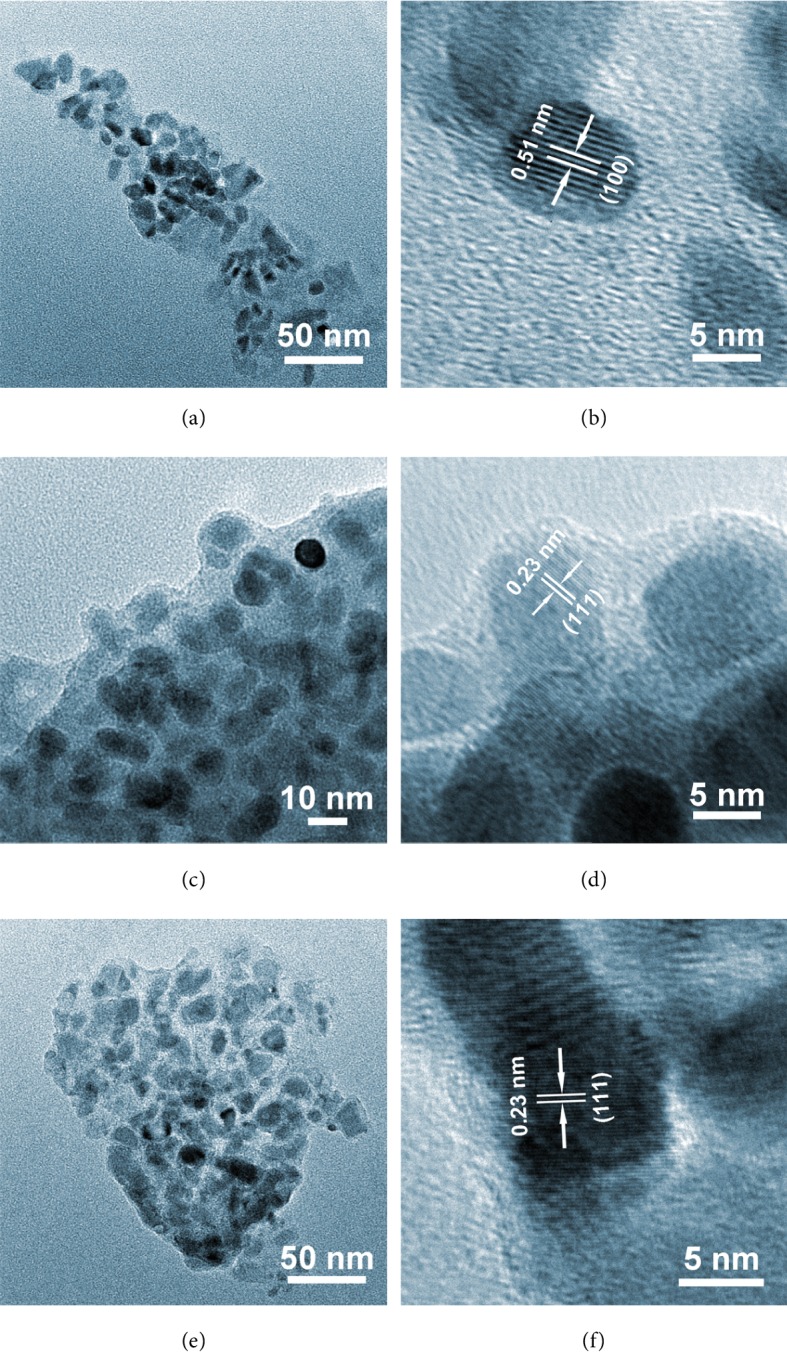
TEM and HRTEM images of (a, b) NP-10k-T3, (c, d) NP-40k-T3, and (e, f) NP-360k-T3.

The detailed structure of these samples was further analyzed under TEM (Figure 2), and it appears that these samples are composed of numerous particles, instead of well-defined one-dimensional structures. The formation of Ni_2_P nanoparticles has been previously obtained by direct phosphorization of Ni foam [27]. Moreover, these nanoparticles appear to be embedded inside a carbonaceous matrix, which is consistent with the above SEM results. The HRTEM images (Figures 2(b), 2(d), and 2(f)) indicate that the nanoparticles of NP-10k-T3, NP-40k-T3, and NP-360k-T3 show some visible lattice fringes with an interplanar distance of about 0.51, 0.23, and 0.23 nm, corresponding to the (100), (111), and (111) planes of Ni_2_P, respectively. As confirmed by X-ray diffraction (XRD; [Supplementary-material supplementary-material-1]), these samples contain a major phase of Ni_2_P (JCPDS No. 74-1385) [28], while some minor phases of CoO (JCPDS No. 75-0533) and NiO (JCPDS No. 75-0197) coexist [29, 30]. The presence of the minor CoO phase could be due to the reduction of Co_3_O_4_ during the hydrothermal step, which is evident in [Supplementary-material supplementary-material-1]. Additionally, it can be noticed that the intensity of the (111) peak increases with the increasing PVP molecular weight, suggesting that better crystallinity was achieved in NP-40k-T3 and NP-160k-T3, which matches well with the previous SEM analysis.

**Figure 3:**
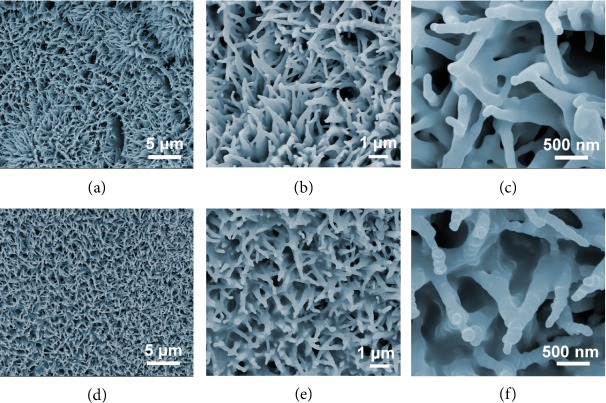
FESEM images of (a–c) NP-10k-T4 and (d–f) NP-10k-T5 at different magnifications.

The morphological evolution for Co_3_O_4_ NWs-Ni at different phosphorization temperatures was further studied. In addition to the previous sample NP-10k-T3 which was treated at 300°C, higher temperatures of 400 and 500°C were also employed in this step, giving rise to samples of NP-10k-T4 and NP-10k-T5, respectively (Figure 3). Compared with NP-10k-T3 (Figures 1(a)–1(c)), these two samples demonstrated much better-defined nanowires wrapped with a smooth outer layer. Furthermore, the film-like structure which was present in NP-10k-T3 becomes almost absent in these two samples, suggesting that any organic compounds (could be derived from PVP or ethylene glycol) are better pyrolyzed at higher temperatures, leaving behind fewer residues.

**Figure 4:**
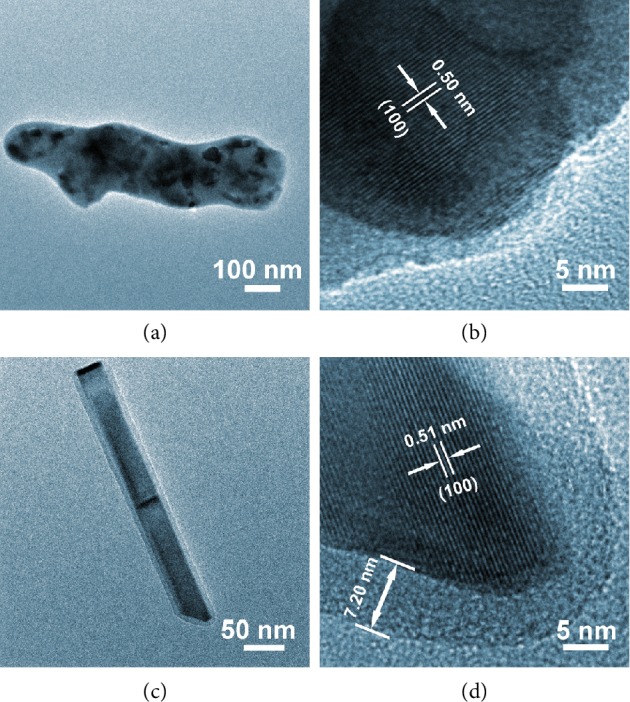
TEM and HRTEM images of (a, b) NP-10k-T4 and (c, d) NP-10k-T5.

Such structures are also viewed under TEM (Figure 4), and instead of small nanoparticles embedded inside the carbonaceous matrix, larger crystallites coated with a thin carbon layer can be observed. The XRD results also confirmed that better crystallinity can be achieved at higher phosphorization temperatures, as the intensity of the (111) peak increases significantly when the temperature increases from 300 to 400°C and to 500°C. Similarly, all the three samples in [Supplementary-material supplementary-material-1] contain a major phase of Ni_2_P with the coexistent CoO and NiO phases. However, another Ni-rich phase of Ni_12_P_5_ is also presented in NP-10k-T5, which could be probably attributed to the phosphorization of the Ni foam to a greater extent at this elevated temperature, giving rise to a transition layer between the Ni foam and the Ni_2_P [31].

The surface element and the valence state of C, Co, Ni, and P in the sample NP-10k-T3 were further detected by X-ray photoelectron microscopy (XPS). As shown in [Supplementary-material supplementary-material-1], the C 1s spectrum is deconvoluted into four peaks located at 284.8, 285.4, 286.3, and 289.1 eV, which can be ascribed to be C-C, C-P, C-O, and O=C–O, respectively [32, 33]. For the spectrum of Co ([Supplementary-material supplementary-material-1]), the peaks at 782.7 and 798.3 eV are assigned to the Co^2+^ in CoO [34, 35]. In [Supplementary-material supplementary-material-1], the peaks at binding energy of 857.4 and 875.4 eV correspond to Ni 2p_3/2_ and Ni 2p_1/2_, respectively, followed by two satellite peaks at 863.7 and 882.0 eV, which probably are characteristic of Ni^*σ*+^ in Ni_2_P [28, 36]. Furthermore, the peaks at 133.2 and 134.0 eV in P 2p ([Supplementary-material supplementary-material-1]) could be attributed to P-C and P-O, respectively [32].

Several control experiments were conducted to further understand the formation of Ni_2_P. It is shown in [Supplementary-material supplementary-material-1] that the nanowires synthesized without PVP are intensively agglomerated, suggesting the important role of PVP as an effective surfactant to maintain the monodispersity of the nanowires. Other two control samples of Co_3_O_4_ NWs-P and Ni foam-P exhibit the similar nanowire structure ([Supplementary-material supplementary-material-1]), with Ni_2_P as their main phase according to the XRD patterns ([Supplementary-material supplementary-material-1]) and a Ni-rich phase of Ni_5_P_4_ present in Ni foam-P [37]. In addition, oxidation of Co_3_O_4_ NWs-Ni at 400°C in air was performed to serve as a comparison to phosphorization, and this sample shows a highly porous nanowire structure with the oxidized phases of Co_3_O_4_ and NiO ([Supplementary-material supplementary-material-1]).

**Figure 5:**
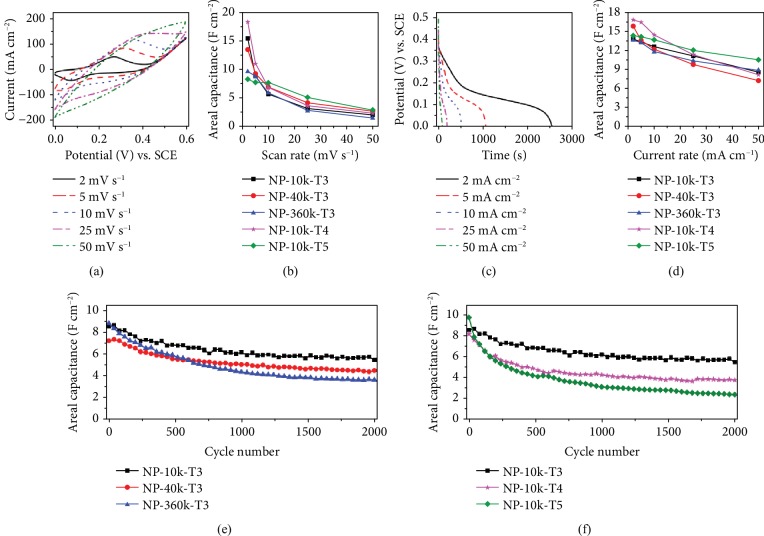
Supercapacitor performance of CoO@Ni_2_P series materials with different average molecular weights of PVP and phosphating temperatures in a three-electrode system: (a) CV curves of NP-10k-T3 at different scan rates. (b) The areal capacitance contrast calculated from corresponding CV curves. (c) Galvanostatic discharge curves of NP-10k-T3 at different current rates. (d) The areal capacitance contrast calculated from corresponding galvanostatic discharge curves. (e, f) Long-term charge-discharge performance contrast at a current rate of 50 mA cm^−2^.

These materials were then utilized as the working electrode for the supercapacitor first in a three-electrode system. Cyclic voltammograms (CVs) at different scan rates for NP-10k-T3 were shown in Figure 5(a), and the results of other samples are shown in [Supplementary-material supplementary-material-1]. In the CVs of all samples, a pair of redox peaks can be distinctly observed, which are ascribed to the reversible reactions of Ni^2+^/Ni^3+^ due to faradic redox reaction of Ni_2_P in basic electrolyte corresponding to the following reaction [22]:

(1) $$\begin{array}{c} {\text{Ni}}^{2+}+2\text{O}{\text{H}}^{–}⟷\text{Ni}{\left(\text{OH}\right)}_{2} \end{array}$$

(2) $$\begin{array}{c} \text{Ni}{\left(\text{OH}\right)}_{2}+{\text{OH}}^{–}⟷\text{NiOOH}+{\text{H}}_{2}\text{O}+e^{–} \end{array}$$

The areal capacitances *C* (F cm^−2^) for these Ni_2_P@carbon materials can be calculated from corresponding CV curves using the following equation [38, 39]:

(3) $$\begin{array}{c} C=\frac{\int I\left(V\right)dV}{v\Delta V}, \end{array}$$

where *I* (A cm^−2^) is the current density, *v* (mV s^−1^) is the scan rate, and Δ*V* (V) is the difference between cathodic and anodic potential. As depicted in Figure 5(b), the areal capacitance of these samples calculated from the CVs is quite similar, and they do not show a significant difference among the samples.

The galvanostatic discharge curves of NP-10k-T3 at different current rates are displayed in Figure 5(c), and the corresponding capacitances for these samples can be attained by using the following equation [40, 41]:

(4) $$\begin{array}{c} C=\frac{I\times \Delta t}{\Delta V}, \end{array}$$

where *C* (F cm^−2^) is the areal capacitance, *I* (A cm^−2^) is the current density, Δ*t* (s) is the discharge time, and Δ*V* (V) is the voltage window for charge and discharge. Similarly, these samples do not show contrasting capacitance values obtained from the discharge curves (Figure 5(d)). In conclusion to the results shown above, the use of PVPs with different molecular weights and different phosphorization temperatures makes little difference on the areal capacitance of Ni_2_P, while all of these samples present remarkably high capacitance at these current densities. This might be due to the intrinsic excellent electrical conductivity and high electrochemical activity of Ni_2_P [22]. Figure 5(e) reveals the cycle stability of the samples synthesized using PVPs with different molecular weights at a constant current rate of 50 mA cm^−2^. Notably, upon 2000 charge-discharge cycles, the NP-10k-T3 could still keep a high capacitance of 5.5 F cm^−2^. However, with the increase in the average molecular weight of PVP, the capacitances of NP-40k-T3 and NP-360k-T3 gradually decrease to 4.5 and 3.6 F cm^−2^, respectively, at the end of tests.

The cycling performances of samples synthesized at different phosphorization temperatures, namely, NP-10k-T4 and NP-10k-T5, were included and compared. As shown in Figure 5(f), the performance of these three samples shows quite contrasting stability, where NP-10k-T3 exhibits the best retention that a stable capacitance of 5.5 F cm^−2^ can be delivered after 2000 cycles. The other two samples of NP-10k-T4 and NP-10k-T5 can only retain capacitances of 3.8 and 2.3 F cm^−2^, respectively. The detailed electrochemical values of these samples are listed and compared in [Supplementary-material supplementary-material-1]. In view of the structure characteristics of these samples, the best performance of NP-10k-T3 could be attributed to the much smaller particles contained in the sample, offering a more efficient charge transfer process.

**Figure 6:**
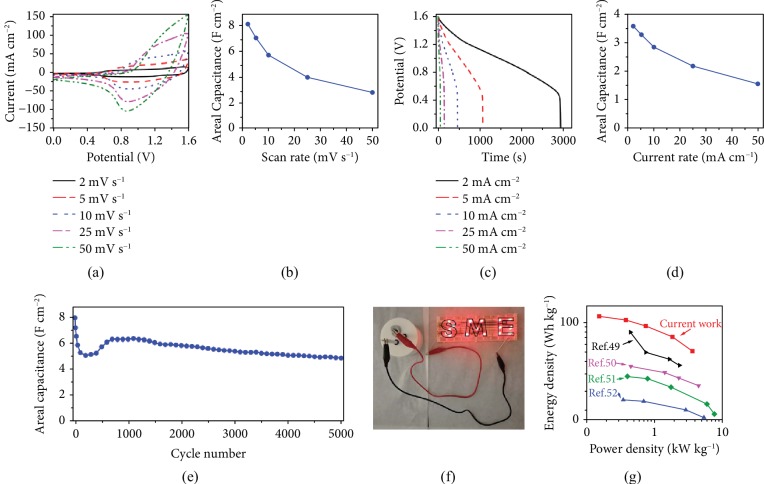
Performance of the NP-10k-T3||AC ASC: (a) CVs at different scan rates and (b) the corresponding capacitance calculated from (a). (c) Galvanostatic discharge curves at different current rates and (d) the corresponding capacitance calculated from (c). (e) Long-term charge-discharge performance at a current rate of 25 mA cm^−2^. (f) A photograph shows an ASC powering up 16 red LEDs arranged in the pattern of “SME.” (g) Ragone plot which compares the energy density versus power density curves of the NP-10k-T3||AC asymmetric supercapacitor.

This hypothesis was confirmed by the electrochemical impedance spectroscopy (EIS) analysis ([Supplementary-material supplementary-material-1]). Based on the fitted data using the equivalent circuit shown in [Supplementary-material supplementary-material-1], NP-10k-T3 indeed shows the lowest charge transfer resistance among the five samples. To shed some light on the stability, the NP-10k-T3 was further characterized by XPS analysis after 2000 charge-discharge cycles in the three-electrode system. As shown in [Supplementary-material supplementary-material-1], the C 1s spectrum was similarly deconvoluted into four peaks located at 284.7, 286, and 289.1 eV, which can be ascribed to be C-C, C-O, and O=C–O, respectively. The peak due to the C-P bond is absent after the cycling test. In the Co 2p region ([Supplementary-material supplementary-material-1]), the peaks at 780.7 and 796.7 eV are assignable to Co 2p_3/2_ and Co 2p_1/2_ attributed to Co^2+^. The Ni 2p in [Supplementary-material supplementary-material-1] shows Ni 2p_3/2_ (855.6 eV) and Ni 2p_1/2_ (873.2 eV), which can be ascribed to the Ni(OH)_2_, indicating the transformation of Ni-P into Ni-OH in the basic electrolyte during the cycling test [42]. Moreover, the P 2p in [Supplementary-material supplementary-material-1] shows a broad peak at 134.5 eV assigned to the P-O bond, while the original feature of the P-C bond disappeared completely. All these observations indicate that the Ni_2_P species in NP-10k-T3 are partially oxidized to nickel oxide/hydroxide on the surface after a long cycling test in basic solution, which then cooperated with Ni_2_P as an efficient supercapacitor material [43]. This observation is in accordance with the recent reports [44, 45]. Additionally, the electrochemical properties of the control materials (Ni foam-P, Co_3_O_4_ NWs, and Co_3_O_4_ NWs-P) were also investigated ([Supplementary-material supplementary-material-1]), and the results further highlight the superior performance of the NP-10k-T3 sample. The advantage of NP-10k-T3 over Co_3_O_4_ NWs-Ni-O_2_ ([Supplementary-material supplementary-material-1]) also suggests that Ni_2_P may be more suitable for electrochemical applications than the NiO counterpart. An asymmetric supercapacitor (ASC) was subsequently assembled with the NP-10k-T3 cathode and activated carbon (AC) anode (NP-10k-T3||AC). After testing the device with different voltage windows ([Supplementary-material supplementary-material-1]), the suitable working voltage window for the NP-10k-T3||AC ASC is chosen as 0–1.6 V.  Figure 6a shows the CV curves at different scan rates where a pair of current peaks at around 0.87 V and 1.3 V can be observed at 50 mV s^−1^. The areal capacitance is calculated to be 8.2, 7.1, 5.7, 4.0, and 2.8 F cm^−2^ from 2 to 50 mV s^−1^ (Figure 6(b)).  Figure 6(c) presents the charge-discharge curves at various current rates, with excellent corresponding discharge capacitances of 3.7, 3.3, 2.9, 2.2, and 1.6 F cm^−2^ obtained at 2, 5, 10, 25, and 50 mA cm^−2^, respectively (Figure 6(d)). For the long-term charge-discharge performance at a current rate of 25 mA cm^−2^ (Figure 6(e)), the capacitance rapidly reduces from 2.0 F cm^−2^ to 1.3 F cm^−2^ for the first 200 cycles, which is quite commonly reported in many supercapacitor studies [15, 46]. It is probably ascribed to unmatched storage capabilities between NP-10k-T3 and activated carbon [47, 48]. The capacitance remains as 1.2 F cm^−2^ after continuous charge and discharge for 5000 times.  Figure 6(f) is a photograph showing that one of the ASC effortlessly powers up 16 red LEDs arranged in the pattern of “SME.”

Figure 6(g) shows the Ragone plots comparing the energy density versus power density of the ASC with other reported supercapacitors. The energy density (*E*) and power density (*P*) of the ASC device are obtained by the following equations:

(5) $$\begin{array}{c} E=\frac{1}{2}C\Delta V^{2}, \end{array}$$

(6) $$\begin{array}{c} P=\frac{E}{\Delta t}, \end{array}$$

where *C* (F g^−1^) is the specific capacity, Δ*V* (V) is the practical potential difference, and Δ*t* is (s) is the discharge time. As a result, the ASC device can reach a high energy density of about 122.8 Wh kg^−1^ at a power density of 0.15 kW kg^−1^, and it remains 53.3 Wh kg^−1^ at the highest power density of 3.78 kW kg^−1^. More significantly, the NP-10k-T3||AC ASC is superior to other nickel phosphide-based supercapacitors, either energy density or power density [49–52]. All of the above results prove the outstanding supercapacitor performance of NP-10k-T3||AC ASC and its potential of practical application in electrochemical energy storage.

## 3. Conclusions and Outlook

In this work, the composite Ni_2_P@carbon directly grown on Ni foam was prepared and applied for the supercapacitor. A series of samples were synthesized using PVPs with different molecular weights and by phosphorization at different temperatures, and they were shown to have a structure containing carbon-coated Ni_2_P nanoparticles supported on the Ni foam substrate. By optimizing these critical conditions, the sample of NP-10k-T3 synthesized using PVP-10k and phosphorized at 300°C demonstrated the best performance with a capacitance of 8.5 F cm^−2^ at a high discharge current rate of 50 mA cm^−2^ and a stable capacitance of 5.5 F cm^−2^ after 2000 charge-discharge cycles. The above excellent electrochemical properties could be attributed to the small Ni_2_P nanoparticles contained in the sample offering highly efficient charge transfer kinetics and the supporting carbonaceous matrix providing good structure stability. More significantly, when an asymmetric supercapacitor (ASC) was assembled with this sample as the cathode and activated carbon (AC) as the anode, the device showed excellent capacitances of 3.7 and 1.6 F cm^−2^ at 2 and 50 mA cm^−2^, respectively, and it kept a high capacitance of 1.2 F cm^−2^ after 5000 cycles at a current rate of 25 mA cm^−2^. In addition, the ASC could deliver a high energy density of about 122.8 Wh kg^−1^ at a power density of 0.15 kW kg^−1^ and 53.3 Wh kg^−1^ at the highest power density of 3.78 kW kg^−1^ and power up 16 red LEDs effortlessly, demonstrating promising potential for practical application.

## Supplementary Materials

Supplementary Materials Figure S1: FESEM images of (a-c) Co NWs, (d-f) Co3O4 NWs, and (g-i) Co3O4 NWs-Ni at different magnifications. Figure S2: XRD patterns of (I) Co3O4 NWs and (II) Co3O4 NWs-Ni. Figure S3: (a) XRD patterns of (I) NP-360k-T3, (II) NP-40k-T3, and (III) NP-10k-T3. (b) XRD patterns of (I) NP-10k-T5, (II) NP-10k-T4, and (III) NP-10k-T3. Figure S4: XPS spectrums of (a) Co 2p, (b) Ni 2p, and (c) P 2p in NP-10k-T3. Figure S5: (a-c) FESEM images of NP-10k-T3 synthesized without PVP at different magnifications. Figure S6: FESEM images of (a-c) Co3O4 NWs-P and (d-f) Ni foam-P at different magnifications. Figure S7: XRD patterns of (a) Co3O4 NWs-P and (b) Ni foam-P. Figure S8: (a-c) FESEM images of Co3O4 NWs-Ni-O_2_ at different magnifications. (d) XRD pattern of Co3O4 NWs-Ni-O_2_. Figure S9: supercapacitor performance of CoO@Ni2P series materials with different average molecular weights of PVP and phosphating temperatures in a three-electrode system. Figure S10: (a) EIS studies of (I) NP-10k-T3, (II) NP-40k-T3, and (III) NP-360k-T3. (b) EIS studies of (I) NP-10k-T3, (II) NP-10k-T4, and (III) NP-10k-T5. (c) The equivalent circuits of five samples from the EIS analysis. Figure S11: XPS spectrums of (a) C 1s, (b) Co 2p, (c) Ni 2p, and (d) P 2p for NP-10k-T3 after 2000 charge-discharge cycles in the three-electrode system. Figure S12: supercapacitor performance of contrast materials in a three-electrode system. Figure S13: supercapacitor performance of Co3O4 NWs-Ni-O_2_ in a three-electrode system. Figure S14: CVs of the NP-10k-T3||AC ASC with different voltage windows at a scan rate of 25 mV s^−1^. Table S1: comparison of performance for different samples of the current work.
