# Supplementary material for: PVP-Assisted Synthesis of Self-Supported Ni2P@Carbon for High-Performance Supercapacitor
Source: Research (Wash D C). 2019 Nov 13;2019:8013285. doi: 10.34133/2019/8013285 (PMC6944484; doi:10.34133/2019/8013285)
Supplement: Supplementary Materials — Figure S1: FESEM images of (a-c) Co NWs, (d-f) Co3O4 NWs, and (g-i) Co3O4 NWs-Ni at different magnifications. Figure S2: XRD patterns of (I) Co3O4 NWs and (II) Co3O4 NWs-Ni. Figure S3: (a) XRD patterns of (I) NP-360k-T3, (II) NP-40k-T3, and (III) NP-10k-T3. (b) XRD patterns of (I) NP-10k-T5, (II) NP-10k-T4, and (III) NP-10k-T3. Figure S4: XPS spectrums of (a) Co 2p, (b) Ni 2p, and (c) P 2p in NP-10k-T3. Figure S5: (a-c) FESEM images of NP-10k-T3 synthesized without PVP at different magnifications. Figure S6: FESEM images of (a-c) Co3O4 NWs-P and (d-f) Ni foam-P at different magnifications. Figure S7: XRD patterns of (a) Co3O4 NWs-P and (b) Ni foam-P. Figure S8: (a-c) FESEM images of Co3O4 NWs-Ni-O2 at different magnifications. (d) XRD pattern of Co3O4 NWs-Ni-O2. Figure S9: supercapacitor performance of CoO@Ni2P series materials with different average molecular weights of PVP and phosphating temperatures in a three-electrode system. Figure S10: (a) EIS studies of (I) NP-10k-T3, (II) NP-40k-T3, and (III) NP-360k-T3. (b) EIS studies of (I) NP-10k-T3, (II) NP-10k-T4, and (III) NP-10k-T5. (c) The equivalent circuits of five samples from the EIS analysis. Figure S11: XPS spectrums of (a) C 1s, (b) Co 2p, (c) Ni 2p, and (d) P 2p for NP-10k-T3 after 2000 charge-discharge cycles in the three-electrode system. Figure S12: supercapacitor performance of contrast materials in a three-electrode system. Figure S13: supercapacitor performance of Co3O4 NWs-Ni-O2 in a three-electrode system. Figure S14: CVs of the NP-10k-T3||AC ASC with different voltage windows at a scan rate of 25 mV s−1. Table S1: comparison of performance for different samples of the current work. [file 8013285.f1.docx]

**Supplementary Materials**

**PVP-assisted Synthesis of Self-supported Ni_2_P@carbon for High-Performance Supercapacitor**

**Qian He,^1^ Xiong Xiong Liu,^1^ Rui Wu,^1^ Jun Song Chen*^12^**

*^1^School of Materials and Energy, University of Electronic Science and Technology of China, Chengdu, 611731, P. R. China.*

*^2^Center for Applied Chemistry, University of Electronic Science and Technology of China, No.2006, Xiyuan Ave. West Hi-Tech Zone, Chengdu, China.*

*Correspondence should be addressed to Jun Song Chen; jschen@uestc.edu.cn

**Experimental section**

1. **Materials**

Cobalt nitrate hexahydrate (Co(NO_3_)·6H_2_O, 99%, Adamas Reagent), Ammonium fluoride (NH_4_F, 99%, Adamas Reagent), Urea (AR, Sinopharm Chemical Reagent), Ni foam (Shenzhen Tianchenghe Technology, China), Polyvinylpyrrolidone (PVP, AR, Aladdin), Ethylene glycol (AR, Chengdu Chron Chemicals, China), Nickel chloride hexahydrate (NiCl_2_·6H_2_O, AR, Sinopharm Chemical Reagent), Ethanol (AR, Chengdu Chron Chemicals, China), Sodium hypophosphite (NaH_2_PO_2_, AR, Aladdin), Sodium hydroxide (NaOH, AR, Chengdu Chron Chemicals, China). Activated carbon (Tianjin Aiweixin Chemicals, China), Carbon black Super-P-Li (Tianjin Aiweixin Chemicals, China) and Poly(vinylidene fluoride) (PVDF, Tianjin Aiweixin Chemicals, China). All the reagents used in the experiment were of analytical grade purity and were used as received.

1. **Sample synthesis**

**2.1 Synthesis of Co_3_O_4_ NWs.** The Co_3_O_4_ NWs grown on Ni foam were synthesized by a simple hydrothermal method followed by calcination [1]. 1 mmol Co(NO_3_)·6H_2_O, 2 mmol NH_4_F and 5 mmol urea were dissolved into 10 ml deionized H_2_O under stirring for 10 min. Afterwards, a slice of cleaned Ni foam (2×6 cm^2^) was put into the above solution and transferred into a Teflon-lined stainless steel autoclave, and then maintained at 120 °C for 5 h. After cooling down to the room temperature, the Co NWs were taken out and flushed with deionized water, and dried at 60 °C overnight. Then the Co NWs were heated at 400 °C in air for 2 h at a heating rate of 2 °C min^-1^. Finally, the sample was obtained and designated as Co_3_O_4_ NWs.

**2.2 Synthesis of Co_3_O_4_ NWs-Ni.** First, 500 mg polyvinylpyrrolidone (PVP, with different molecular weight) were dissolved into 25 ml ethylene glycol under stirring and heating. Then 3.2 ml ethylene glycol containing 0.64 mmol NiCl_2_·6H_2_O was poured slowly into the above solution under stirring. Finally, the homogeneously mixed solution with a piece of Co_3_O_4_ NWs (2×2 cm^2^) were transferred into a Teflon-lined stainless steel autoclave, and maintained at 160 °C for 12 h. After cooling down naturally, the as-prepared sample was flushed with ethanol and deionized water, and then dried at 60 °C overnight. This sample was designated as Co_3_O_4_ NWs-Ni.

**2.3 Phosphorization of Co_3_O_4_ NWs-Ni.** A piece of Co_3_O_4_ NWs-Ni (1×2 cm^2^) and 400 mg NaH_2_PO_2_ were placed at the heating zone of a tube furnace. NaH_2_PO_2_ was at the upstream side of the furnace, and Co_3_O_4_ NWs-Ni at downstream. Subsequently, the sample was maintained at 300 °C for 2 h at a ramping temperature rate of 2 °C min^-1^ in Ar atmosphere. The phosphorized product was obtained after cooling down to room temperature under Ar. Products synthesized in the system were denoted according to the weight of the PVP used and the phosphorization temperature. For example, the nickel phosphide sample synthesized with PVP-10k and phosphorized at 300 ^o^C is designated as NP-10k-T3. As such, other samples such as NP-40k-T3 and NP-360k-T3 were synthesized using PVP with different molecular weights of 40k and 360k at a common phosphorization temperature of 300 ^o^C, respectively; samples such as NP-10k-T4 and NP-10k-T5 where synthesized with PVP-10k at phophorization temperatures of 400 ^o^C and 500 ^o^C, respectively.

**2.4 Synthesis of Co_3_O_4_ NWs-Ni-O_2_**. A piece of Co_3_O_4_ NWs-Ni was maintained at 400 °C in air for 2 h at a ramping temperature rate of 2 °C min^-1^ to obtain the sample of Co_3_O_4_ NWs-Ni-O_2_.

**2.5 Synthesis of Co_3_O_4_ NWs-P.** The sample was obtained by direct phosphorization of Co_3_O_4_ NWs using the method described above.

**2.6 Synthesis of Ni foam-P.** The sample was obtained by direct phosphorization of bare Ni foam using the method described above.

1. **Material characterization**

A field emission scanning electron microscope (FESEM; FEI Inspect F50) and a high resolution transmission electron microscope (HRTEM; JEM2010F) were employed to observe the morphology of the as-prepared samples. The crystallographic information of the samples were also studied by X-ray diffraction (XRD; Bruker, D8 Advancer; Cu Kα, λ = 1.54 Å). The X-ray photoelectron spectroscopy (XPS) analysis was performed on an Escalab 250Xi XPS.

1. **Electrochemical measurements**

A three-electrode system with the nickel phosphide samples as the working electrode, a Pt wire as the counter electrode and a SCE as the reference electrode in 6 M NaOH aqueous solution was set up for the electrochemical tests, which were carried out on a Bio-logic VMP3-128 electrochemical workstation. The cyclic voltammetry (CV) curves were obtained with a potential window of 0-0.6 V (vs. SCE) at the scan rate of 2, 5, 10, 25 and 50 mV s^-1^. The galvanostatic charge-discharge tests were performed at the different current rates of 2, 5, 10, 25 and 50 mA cm^-2^. The electrochemical impedance spectroscopy (EIS) was conducted within the frequency range from 100 kHz to 0.01 Hz. For the assembling of an asymmetric supercapacitor, a 70:20:10 (wt%) mixture of activated carbon, carbon black Super-P-Li and poly(vinylidene fluoride) (PVDF) was prepared and pasted on Ni foam as the anode.

**Reference**

[1] X. X. Liu, R. Wu, Y. Wang et al., "Self-supported core/shell Co_3_O_4_@Ni_3_S_2_ nanowires for high-performance supercapacitors," *Electrochimica Acta*, vol. 311, pp. 221-229, 2019.


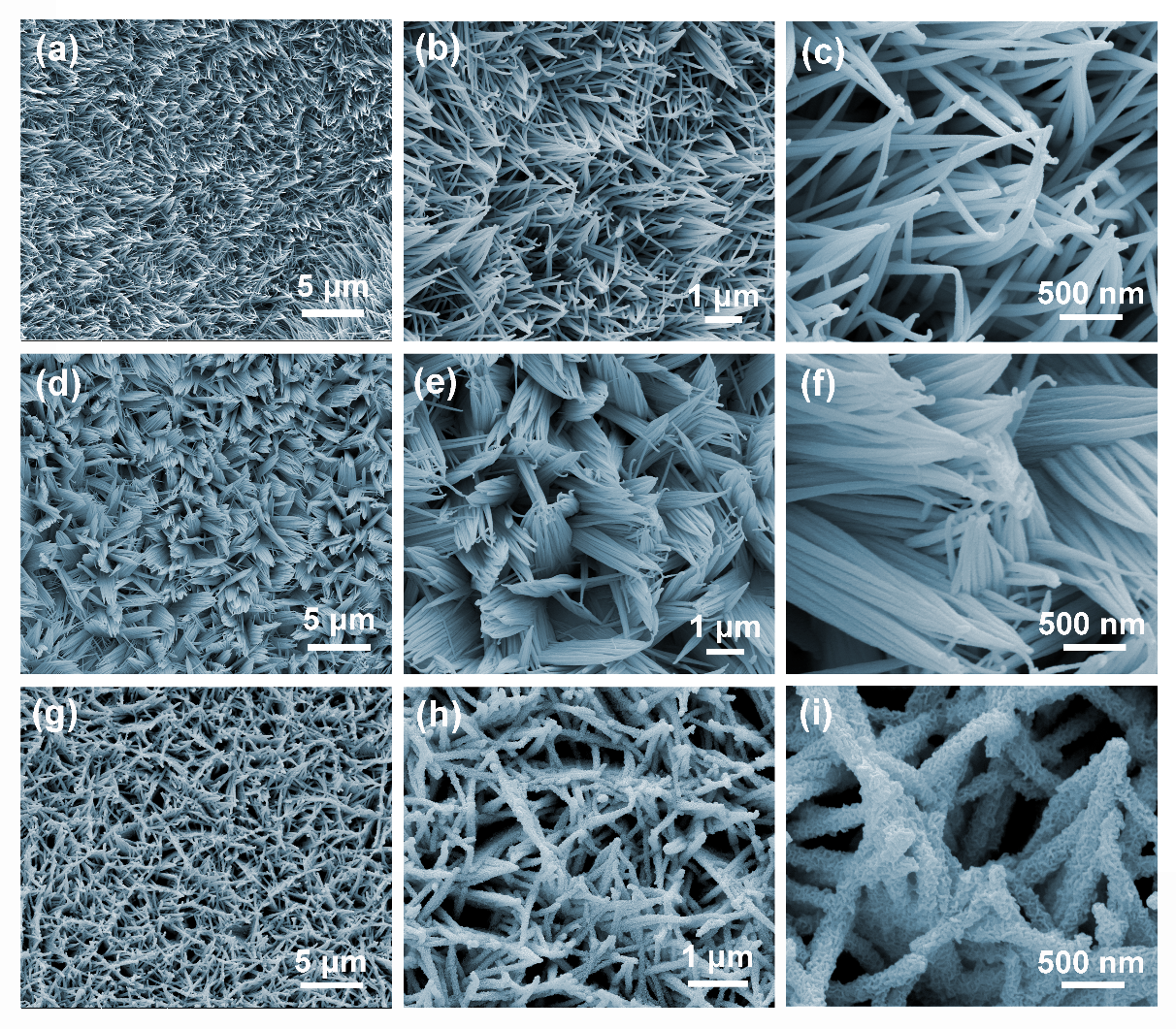


**Figure S1.** FESEM images of (a-c) Co NWs, (d-f) Co_3_O_4_ NWs and (g-i) Co_3_O_4_ NWs-Ni at different magnifications. It is clear that the Co NWs are uniformly distributed on Ni foam and they are in a state of comparative dispersion with smooth surface in (Figure S1a-c). After annealed at 400 °C, the Co_3_O_4_ NWs become clusters of several nanowires compared with Co nanowires and subsequently are served as the skeleton for the further growth of the nickel complex with the addition of PVP (Figure S1d-f). The intermediate product Co_3_O_4_ NWs-Ni still exhibit the major feature of nanowires and considerable crumpled sheets which are considered to be nickel-ethylene glycol complex is covered on to the Co_3_O_4_ NWs (Figure S1g-i).





**Figure S2.** XRD patterns of (I) Co_3_O_4_ NWs, (II) Co_3_O_4_ NWs-Ni. The asterisks mark the peaks corresponding to the Ni foam (JCPDS No. 70-0989). Curve I manifests the Co_3_O_4_ NWs are consisting of Co_3_O_4_ (JCPDS No. 74-2120), CoO (JCPDS No. 75-0533) and NiO (JCPDS No. 75-0197) with Co_3_O_4_ as the major phase. Accordingly, Co_3_O_4_ and CoO are derived from the annealing of Co NWs, and NiO might come from oxidation of a handful of Ni foam. Curve II shows that the Co_3_O_4_ NWs-Ni is mainly composed of CoO as well as a fraction of NiO.


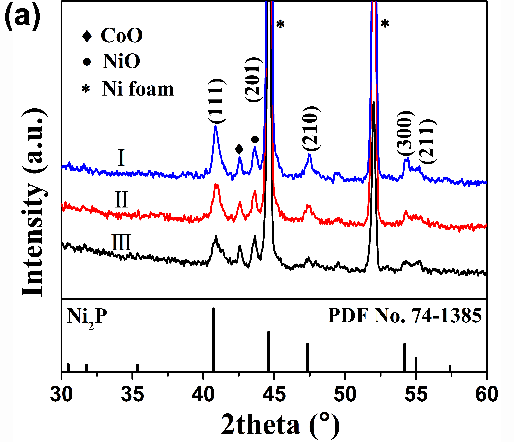

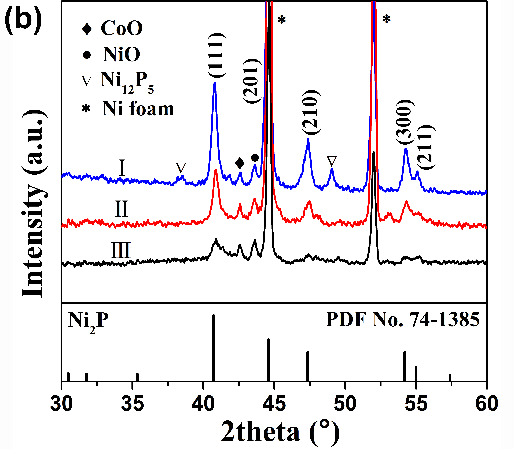


**Figure S3.** (a) XRD patterns of (I) NP-360k-T3, (II) NP-40k-T3 and (III) NP-10k-T3. (b) XRD patterns of (I) NP-10k-T5, (II) NP-10k-T4 and (III) NP-10k-T3. The asterisks in (a) and (b) mark the peaks corresponding to the Ni foam (JCPDS No. 70-0989).


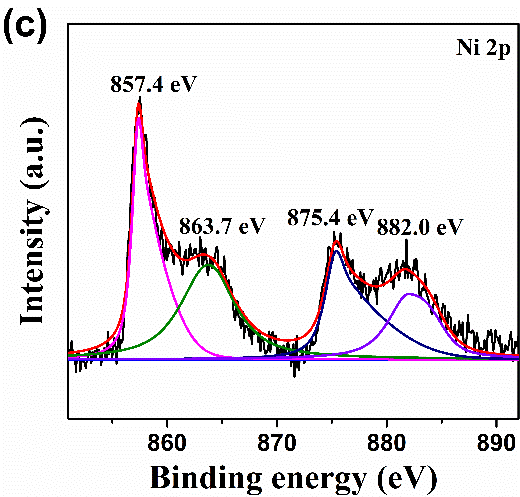

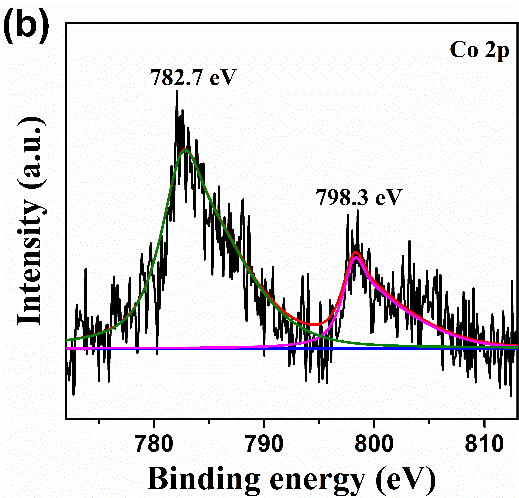

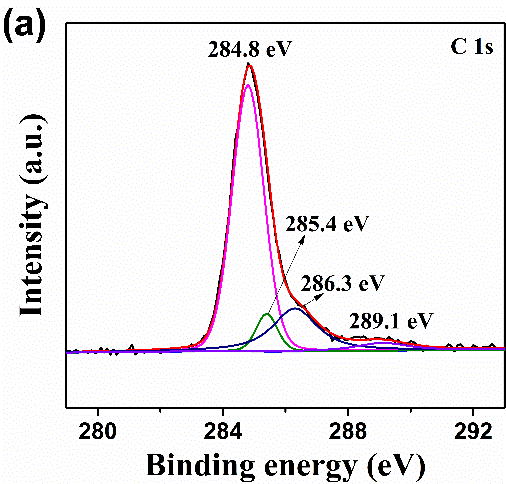

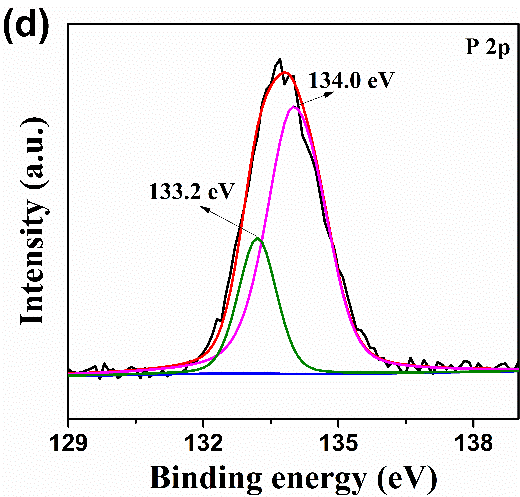


**Figure S4.** XPS spectrums of NP-10k-T3 in the (a) C 1s, (b) Co 2p, (c) Ni 2p and (d) P 2p.

**
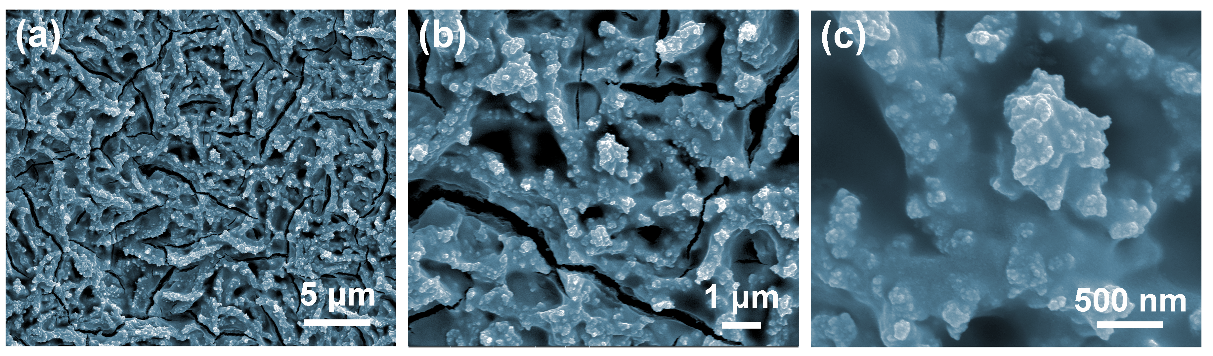
**

**Figure S5.** (a-c) FESEM images of NP-10k-T3 synthesized without PVP at different magnifications. It is apparent that the nickel complex randomly grows on original nanowires and even transforms the “wires” into “blocks”, which would obviously hinder the penetration of electrolyte.


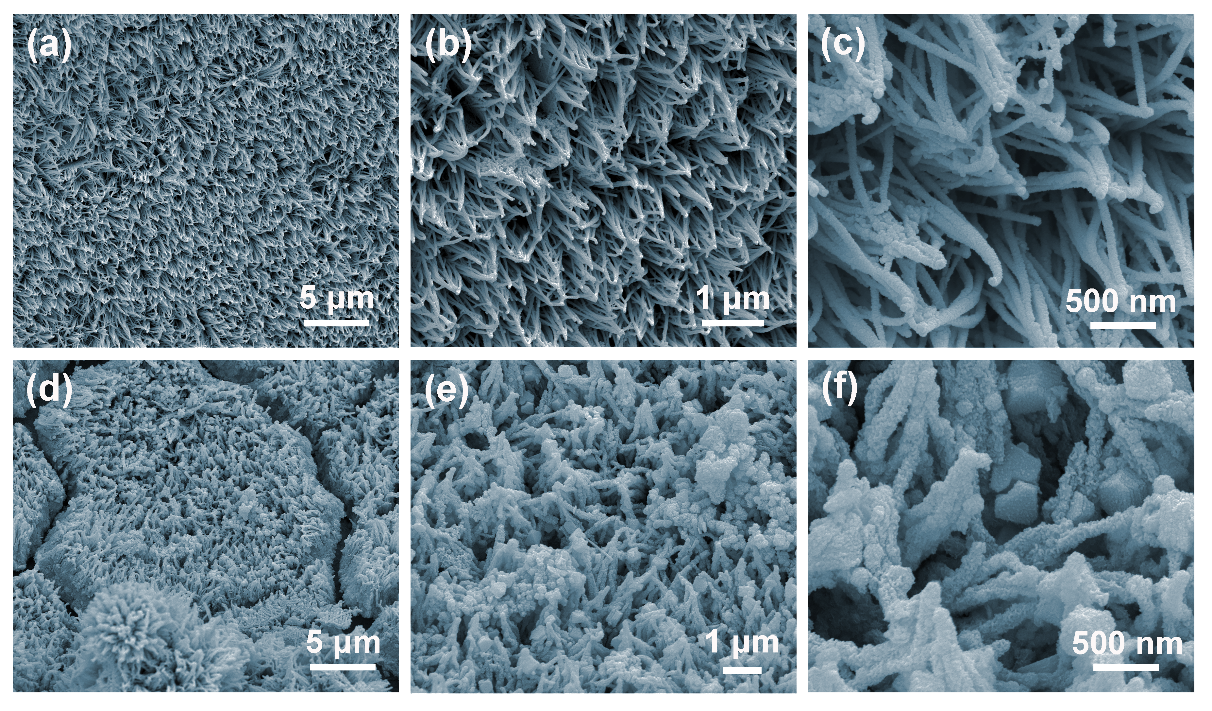


**Figure S6.** FESEM images of (a-c) Co_3_O_4_ NWs-P and (d-f) Ni foam-P at different magnifications. It is clear that the Co_3_O_4_ NWs-P still take nanowires as the main framework, and nanoparticles with uneven distribution adhere on them. While the Ni foam-P display a short nanowire cluster structure and some nanoparticles with non-uniform particle size and random distribution.


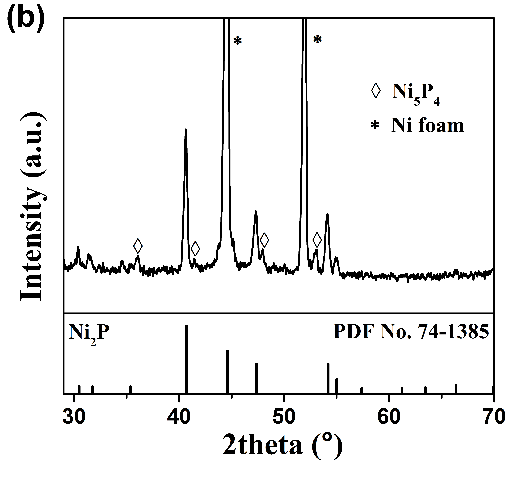

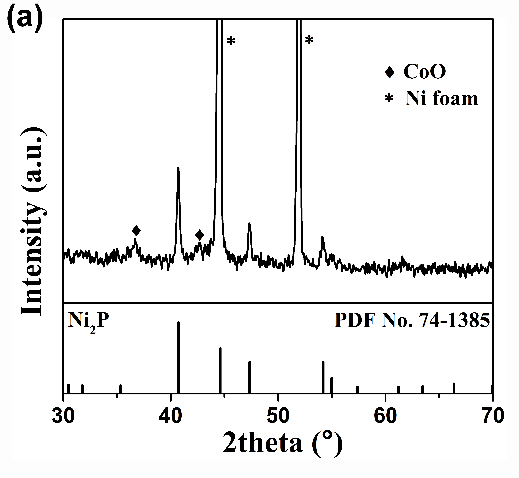


**Figure S7.** XRD patterns of (a) Co_3_O_4_ NWs-P and (b) Ni foam-P. The asterisks in (a) and (b) mark the peaks corresponding to the Ni foam (JCPDS No. 70-0989). It is observed from the figure that the Co_3_O_4_ NWs-P shows strong peaks of Ni_2_P (JCPDS No. 74-1385) and two weak peaks of CoO (JCPDS No. 75-0533). Additionally, the Ni foam-P indicates the two phases of Ni_2_P and Ni_5_P_4_ (JCPDS No. 89-2588).


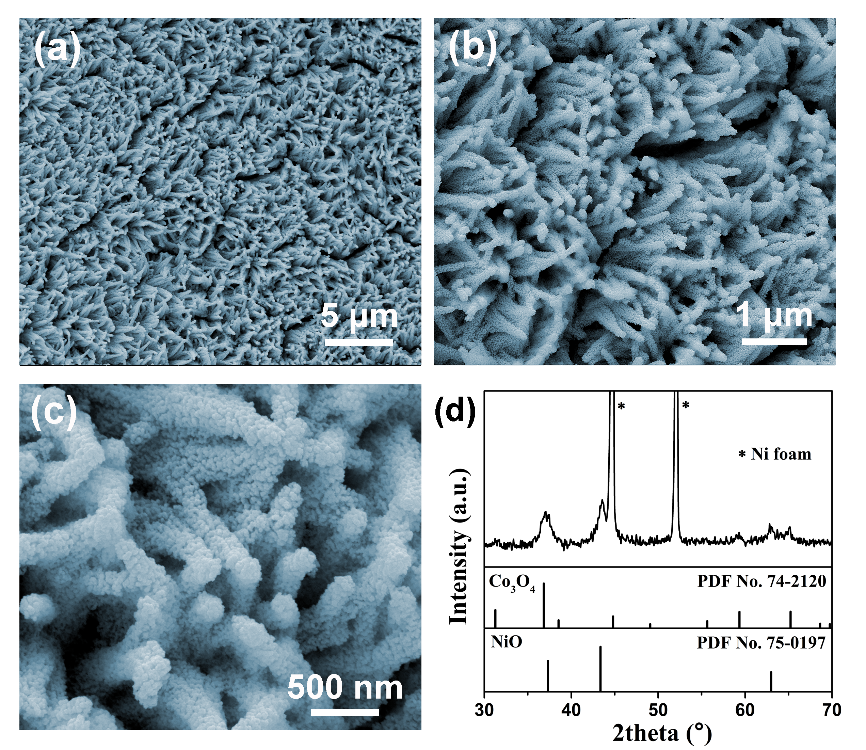


**Figure S8.** (a-c) FESEM images of Co_3_O_4_ NWs-Ni-O_2_ at different magnifications. It is obvious from the images that the Co_3_O_4_ NWs-Ni-O_2_ comprises configuration of the nanowires and numerous nanoparticles wrapping on them. (d) XRD pattern of Co_3_O_4_ NWs-Ni-O_2_. The asterisks mark the peaks corresponding to the Ni foam (JCPDS No. 70-0989). It is apparent that the Co_3_O_4_ NWs-Ni-O_2_ shows two phases of Co_3_O_4_ (JCPDS No. 74-2120) and NiO (JCPDS No. 75-0197). The reappearance of Co_3_O_4_ is probably due to the oxidation of CoO.


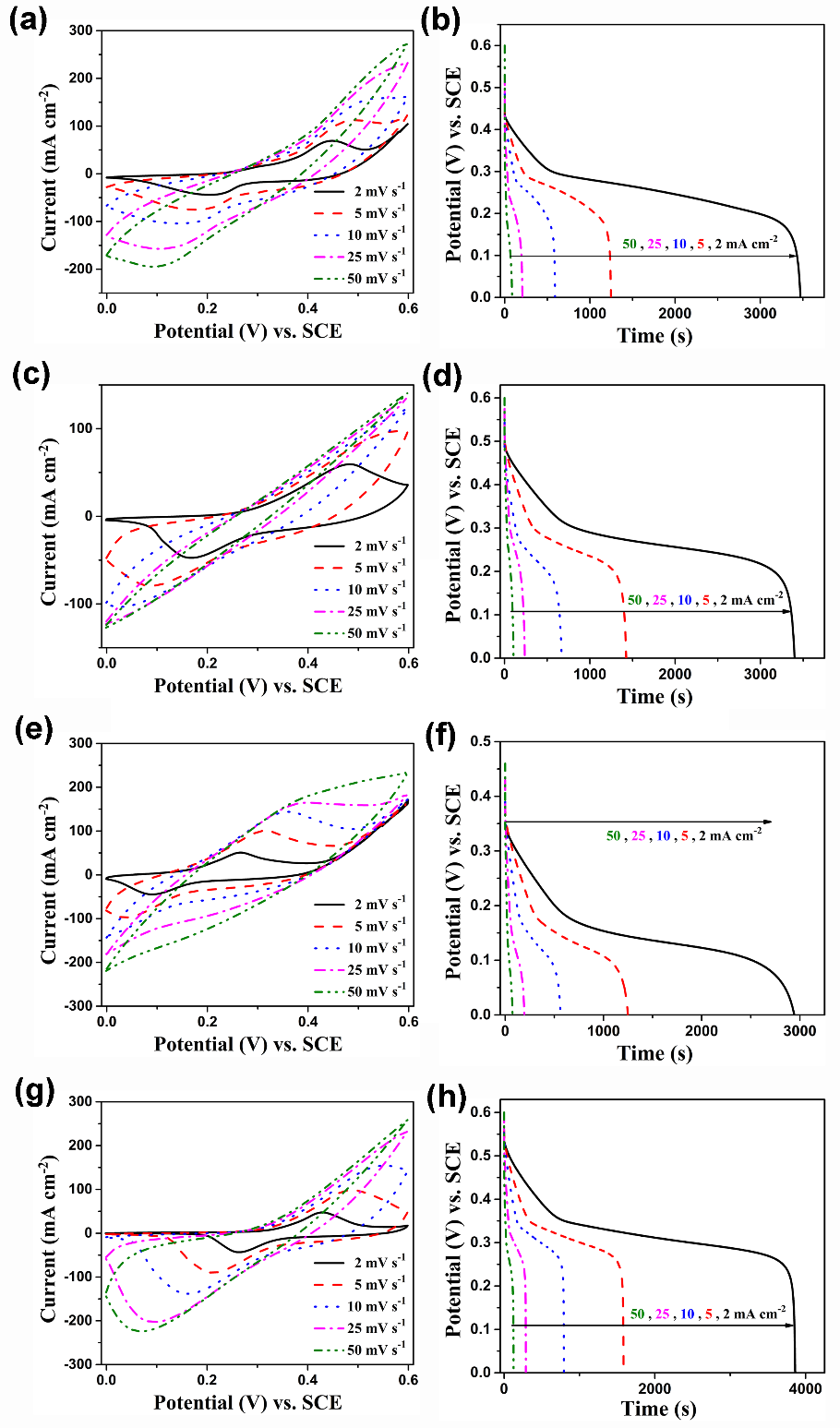


**Figure S9.** Supercapacitor performance of CoO@Ni_2_P series materials with different average molecular weights of PVP and phosphating temperatures in a three-electrode system: (a, c, e and g) CV curves of NP-40k-T3, NP-360k-T3, NP-10k-T4 and NP-10k-T5 at different scan rates, respectively. (b, d, f and h) Galvanostatic discharge curves of NP-40k-T3, NP-360k-T3, NP-10k-T4 and NP-10k-T5 at different current rates, respectively.


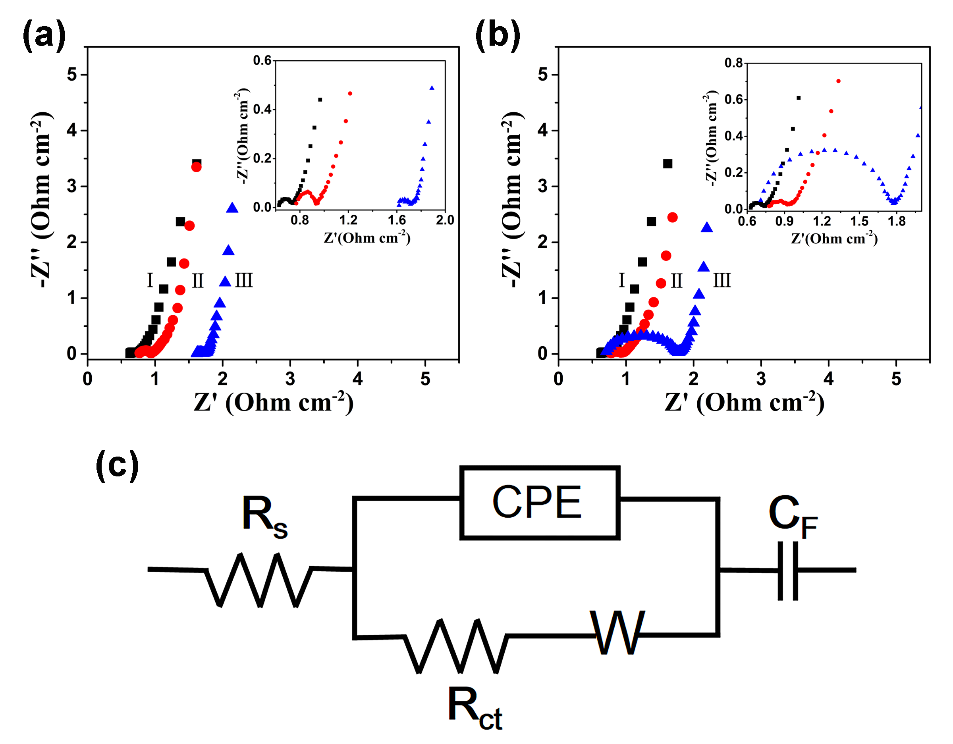


**Figure S10.** (a) EIS studies of (I) NP-10k-T3, (II) NP-40k-T3 and (III) NP-360k-T3. (b) EIS studies of (I) NP-10k-T3, (II) NP-10k-T4 and (III) NP-10k-T5. The inset in (a) and (b) show the zoom-in view of the high frequency range. (c) The equivalent circuits of five samples from the EIS analysis. Particularly, R_s_ represents the equivalent series resistance contains the uncompensated solution resistance, interface resistance and the electronic resistance of the Ni foam, depending on the intercept at the real axis. R_ct_ is the faradaic charge-transfer resistance at the electrode/electrolyte for the redox reactions that based on the diameter of the semicircle.


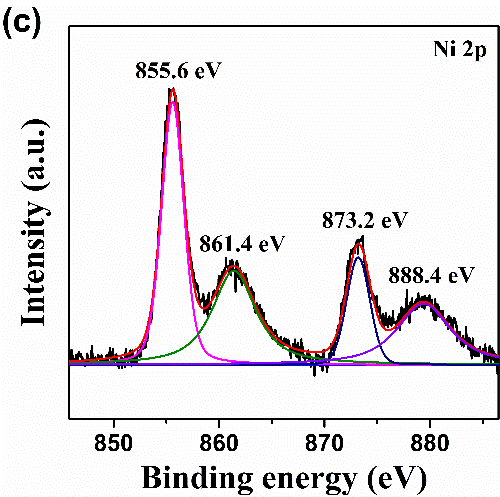

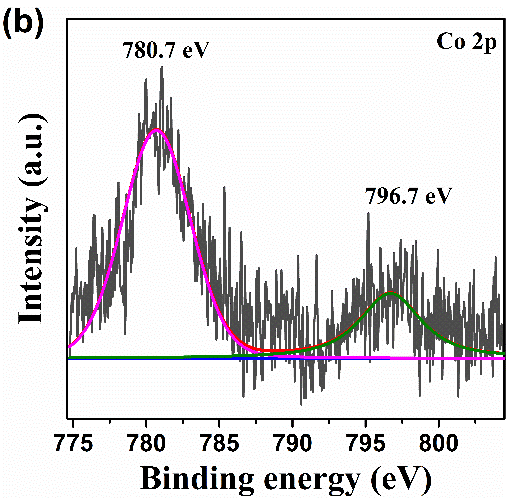

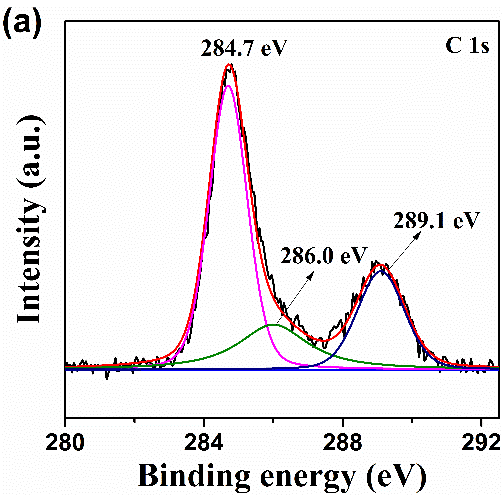

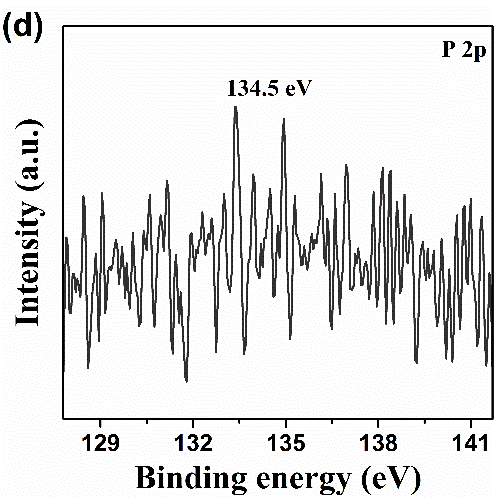


**Figure S11.** XPS spectrums of (a) C 1s, (b) Co 2p, (c) Ni 2p and (d) P 2p for NP-10k-T3 after 2000 charge-discharge cycles in the three-electrode system.


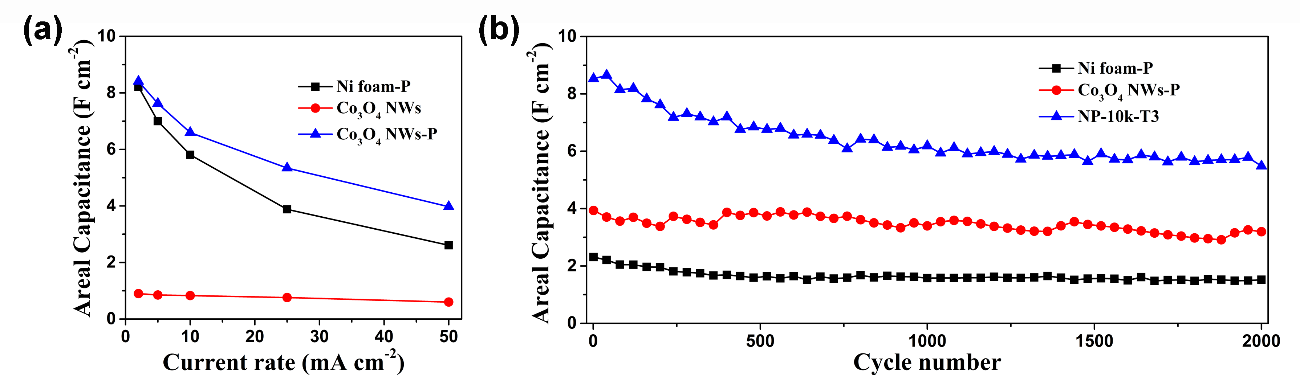


**Figure S12.** Supercapacitor performance of contrast materials in a three-electrode system: (a) The areal capacitance contrast calculated from corresponding galvanostatic discharge curves. The areal capacitances obtained from corresponding galvanostatic discharge curves at the current rates of 2, 5, 10, 25 and 50 mA cm^-2^ of Ni foam-P are 8.2, 7.0, 5.8, 3.9 and 2.6 F cm^-2^; afterwards, the capacitances of Co_3_O_4_ NWs are as low as 0.9, 0.85, 0.83, 0.76 and 0.6 F cm^-2^; finally, the capacitances of Co_3_O_4_ NWs-P are 8.4, 7.6, 6.6, 5.3 and 4.0 F cm^-2^. (b) Long term charge-discharge performance contrast at a current rate of 50 mA cm^-2^. Both of the decay in capacitances of Ni foam-P and Co_3_O_4_ NWs-P is gentle and they maintain at 1.5 and 3.2 F cm^-2^ after 2000 charge-discharge cycles, respectively.


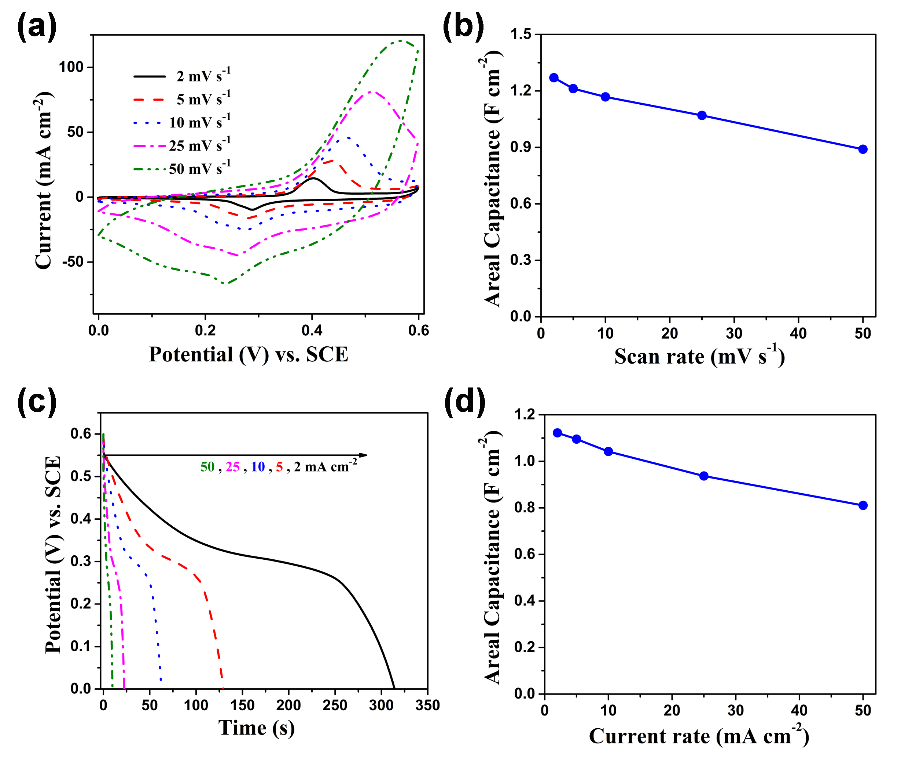


**Figure S13.** Supercapacitor performance of Co_3_O_4_ NWs-Ni-O_2_ in a three-electrode system: (a) CV curves at different scan rates and (b) The corresponding capacitance calculated from (a). (c) Galvanostatic charge-discharge curves at different current rates and (d) the corresponding capacitance calculated from (c). The CVs of Co_3_O_4_ NWs-Ni-O_2_ (Figure S12(a)) show lower oxidation and reduction peak current density compared with NP-10k-T3, corresponding to lower capacitances of 1.3, 1.2, 1.2, 1.1 and 0.9 F cm^-2^ from 2 to 50 mV s^-1^ (Figure S12(b)). Figure S12c shows the galvanostatic charge-discharge curves at 2, 5, 10, 25 and 50 mA cm^-2^, respectively, corresponding to 1.2, 1.1, 1.0, 0.9 and 0.8 F cm^-2^ (Figure S12(d)), noticeably lower than those of NP-10k-T3.





**Figure S14.** CVs of the NP-10k-T3||AC ASC with different voltage windows at a scan rate of 25 mV s^-1^.

**Table S1** Comparison of performance for different samples of current work.

| Electrode materials | Current density/ mA cm^-2^ | Areal capacitance/ F cm^-2^ | Areal capacitance after 2000 cycles at 50 mA cm^-2^/F cm^-2^ |
| --- | --- | --- | --- |
| NP-10k-T3 | 2 | 13.8 | 5.5 |
|  | 50 | 8.5 |  |
| NP-40k-T3 | 2 | 15.8 | 4.5 |
|  | 50 | 7.2 |  |
| NP-360k-T3 | 2 | 13.6 | 3.6 |
|  | 50 | 8.8 |  |
| NP-10k-T4 | 2 | 16.8 | 3.8 |
|  | 50 | 8.1 |  |
| NP-10k-T5 | 2 | 14.3 | 2.3 |
|  | 50 | 10.5 |  |
| Co_3_O_4_ NWs | 2 | 0.9 | / |
|  | 50 | 0.6 |  |
| Co_3_O_4_ NWs-Ni-O_2_ | 2 | 1.1 | / |
|  | 50 | 0.8 |  |
| Co_3_O_4_ NWs-P | 2 | 8.4 | 3.2 |
|  | 50 | 4.0 |  |
| Ni foam-P | 2 | 8.2 | 1.5 |
|  | 50 | 2.6 |  |
